# Microbiota changes induced by microencapsulated sodium butyrate in patients with inflammatory bowel disease

**DOI:** 10.1111/nmo.13914

**Published:** 2020-05-31

**Authors:** Sonia Facchin, Nicola Vitulo, Matteo Calgaro, Andrea Buda, Chiara Romualdi, Daniel Pohl, Barbara Perini, Greta Lorenzon, Carla Marinelli, Renata D’Incà, Giacomo Carlo Sturniolo, Edoardo Vincenzo Savarino

**Affiliations:** ^1^ Department of Surgery, Oncology and Gastroenterology (DISCOG) University Hospital of Padua Padua Italy; ^2^ Department of Biotechnology University of Verona Verona Italy; ^3^ Department of Biology University of Padua Padua Italy; ^4^ Department of Gastroenterology University Hospital Zurich Zurich Switzerland

**Keywords:** 16S metabarcoding, gut inflammation, inflammatory bowel disease, prebiotics, short‐chain fatty acid

## Abstract

**Background:**

Butyrate has shown anti‐inflammatory and regenerative properties, providing symptomatic relief when orally supplemented in patients suffering from various colonic diseases. We investigated the effect of a colonic‐delivery formulation of butyrate on the fecal microbiota of patients with inflammatory bowel diseases (IBDs).

**Methods:**

In this double‐blind, placebo‐controlled, pilot study, 49 IBD patients (n = 19 Crohn's disease, CD and n = 30 ulcerative colitis, UC) were randomized to oral administration of microencapsulated‐sodium‐butyrate (BLM) or placebo for 2 months, in addition to conventional therapy. Eighteen healthy volunteers (HVs) were recruited to provide a healthy microbiota model of the local people. Fecal microbiota from stool samples was assessed by 16S sequencing. Clinical disease activity and quality of life (QoL) were evaluated before and after treatment.

**Key Results:**

At baseline, HVs showed a different microbiota composition compared with IBD patients. Sodium‐butyrate altered the gut microbiota of IBD patients by increasing bacteria able to produce SCFA in UC patients (*Lachnospiraceae* spp.) and the butyrogenic colonic bacteria in CD patients (*Butyricicoccus*). In UC patients, QoL was positively affected by treatment.

**Conclusions and Inferences:**

Sodium‐butyrate supplementation increases the growth of bacteria able to produce SCFA with potentially anti‐inflammatory action. The clinical impact of this finding requires further investigation.

## Graphical abstract

We evaluated the effect of a sodium butyrate microencapsulated oral formulation on the gut microbiota of IBD patients. The butyrate can modulate the gut bacteria, stimulating the growth of butyrogenic and SCFA genera.

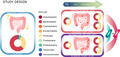

**Key Points**

- Butyrate is important for intestinal health showing anti-inflammatory and regenerative properties. We evaluated the effect of a sodium-butyrate-microencapsulated oral formulation on the gut microbiota of IBD patients.
- Our study showed for the first time that butyrate administration seems to promote the growth of bacteria able to increase the production of butyrate.
- Exogenous butyrate can modulate the gut bacteria, stimulating the growth of butyrogenic and SCFA genera, which in turn may produce more endogenous butyrate for the restoration of intestinal homeostasis.

## 1 INTRODUCTION

Inflammatory bowel diseases (IBDs), including Crohn's disease (CD) and ulcerative colitis (UC), are a group of heterogeneous, chronic, and inflammatory disorders characterized by a deregulated mucosal immune response to commensal gut flora in genetically susceptible individuals exposed to environmental risk factors. Recently, thanks to the advancements of microbiota characterization, the role of dysbiosis in IBD pathogenesis has been emphasized, with different studies showing a reduction in α‐ and β‐diversity.^1^, ^2^

Short‐chain fatty acids (SCFA) represent the final product of saccharolytic fermentation of complex and non‐digestible polysaccharides by anaerobic bacteria.^3^ The main SCFA are acetate, propionate, and butyrate, which are present in the human intestine and depend on diet, site of fermentation, and composition of the intestinal microbiota. Moreover, through a mechanism called *cross‐feeding*,^4^ some bacteria can convert the various SCFA between them. In fecal and mucosal samples from IBD patients, a decrease in butyrogenic colonic bacteria has been found, in particular for some bacteria included in the XIVa and IV clusters, such as *Faecalibacterium prausnitzii* in CD and *Roseburia hominis* in UC.^5^, ^6^

Butyrate is important for intestinal health. In addition to regulating motility, pH and blood flow in the colon and improving the function of mucosal and epithelial intestinal barrier. Moreover, it has antioxidant, antineoplastic, anti‐inflammatory,^7^ and antimicrobial^8^ properties and represents an important energy source for colonocytes. Butyrate can be synthesized from butyryl‐CoA by two different enzymes: butyrate kinase and butyryl‐CoA:acetate CoA‐transferase [BCoAT], the latter being predominant in the human colonic ecosystem.^9^ BCoAT gene content has been shown to be significantly lower in CD subjects compared with healthy controls and UC, suggesting a genetic microbial inability to produce butyrate in CD subjects.^2^ For this reason, butyrate has been employed in some randomized clinical trials and interventional studies to prove its effectiveness in relieving symptoms, especially in diseases with underlying inflammation.^10^ However, data from these studies did not provide conclusive results due to several drawbacks (ie, small sample size, lack of randomization, unclear enrolment criteria, different endpoints, choice of administration route, and difficulties of providing adequate concentrations of butyrate in the colon).^11^, ^12^, ^13^, ^14^, ^15^, ^16^, ^17^, ^18^, ^19^, ^20^, ^21^ Indeed, in the past, butyrate has been administered in the form of enemas in UC,^19^ and in the form of oral tablets in CD,^13^ with low diffusion capacity into the intestinal surface. Moreover, data on the effectiveness of Butyrate on gut microbiota are lacking.

Recently, a new butyrate oral formulation (ButyroseR Lsc Microcaps‐BLM) has been developed. Here, butyrate is contained in a lipophilic microcapsule that provides extensive capacity for intestinal diffusion and facilitates slow release of the active ingredient. This allows subsequent absorption even in the distal portion of the colon.^22^ We decided to perform a pilot, monocentric, prospective, and randomized placebo‐controlled study to evaluate the modulation of the gut microbial composition after butyrate treatment in a group of IBD patients. As secondary aim, the potential effect on clinical activity, fecal calprotectin (FC) levels, and quality of life was also investigated.

## 2 MATERIALS AND METHODS

### 2.1 Intervention compound

A new oral formulation of sodium‐butyrate (Butyrose^®^ Lsc Microcaps‐EP2352386B1, BLM, Sila Srl), at the dose of 3 capsules/d (1800 mg/d), was administered, during the main meals, in consecutive IBD patients, for 60 days. At the same time, a control group received three starch capsules with similar color, flavor, and size.

### 2.2 Ethical statement

A pilot, monocentric, placebo‐controlled randomized study was conducted in accordance with the declaration of Helsinki at the University of Padua from May 2017 to May 2018. The study was approved by the Regional Ethical Committee for Clinical Trials (n. 4049/AO/17). Written informed consent was obtained from all eligible participants before participation.

### 2.3 Subjects and samples

Consecutive patients, aged >18 years, with histologically confirmed diagnosis of CD or UC in the last 6 months and undergoing follow‐up colonoscopy were eligible for the study. The exclusion criteria were (a) prior proctocolectomy; (b) presence of IBD extraintestinal manifestation; (c) treatment with antibiotics in the last 60 days; (d) extensive surgical resection; and (e) presence of stoma. The study coordinator generated the allocation sequence and enrolled the participants. A nurse not involved in the study assigned participants to interventions. Enrolled patients who accepted to participate were randomized in a 1:1 ratio to receive either butyrose (BLM) or placebo (PBO), 3 capsules/d, for 60 days. Allocation was concealed, and all the analysis as well as the clinical and microbiota assessment has been blindly performed to the condition of the patients and to the therapy/placebo assumed. Disease activity was determined by endoscopy, clinical scores, and fecal calprotectin (FC) levels. The FC analysis is routinely performed in IBD patients, and the cutoff referred to the literature.^23^, ^24^ Clinical and endoscopic activity was scored according to the full Mayo score for UC^25^ and the Harvey‐Bradshaw index for CD^26^ with the Simple Endoscopic Score for Crohn's Disease (SES‐CD) for CD.^27^ The localization of the disease was scored according to Montreal classification.^28^ During endoscopy biopsies were obtained according to current guidelines. All patients provided clinical and demographic information and completed the IBDQ questionnaire^29^ at study entry and at the end of follow‐up visit (after 60 days). We collected stool samples from all study participants to analyze the microbiota profile and FC levels, at baseline and after study treatment (after 60 days). During the study, patients were asked to continue their current therapy and diet, and any variation made according to physician judgment on the day of endoscopy was recorded. All the data were collected and located in a password‐protected file. Eighteen healthy volunteers (HVs) were recruited to provide a healthy microbiota model of the local people.^30^ They were asked to provide a single stool sample for fecal microbiota and FC analysis.

### 2.4 Illumina 16S library construction

The stool samples were solubilized and stabilized by degradation in Xpedition Buffer (Zymo Research) and stored at −20°C until the analysis. Sequencing protocol was performed at BMR Genomics srl. Briefly: V3–V4 regions of 16S rRNA gene were amplified using the primers Pro341F: 5′‐CCTACGGGNBGCASCAG‐3′ and Pro805R: Rev 5′‐GACTACNVGGGTATCTAATCC‐3′.^31^ Primers were modified with forward overhang: 5′‐TCGTCGGCAGCGTCAGATGTGTATAAGAGACAG [locus‐specific sequence]‐3′ and with reverse overhang: 5′‐GTCTCGTGGGCTCGGAGATGTGTATAAGAGACAG [locus‐specific sequence]‐3′ necessary for dual‐index library preparation, following Illumina protocol https://web.uri.edu/gsc/files/16s‐metagenomic‐library‐prep‐guide‐15044223‐b.pdf. Samples were normalized, pooled, and run on Illumina MiSeq with 2 × 300 bp approach.

### 2.5 Bioinformatics analyses

The fastq sequences were analyzed using DADA2,^32^ a new tool that implements an error correction model and allows to identify exact sample sequences that differ as little as a single nucleotide. The final output of DADA2 is an amplicon sequence variant (ASV) table which records the number of times each exact amplicon sequence variant was observed in each sample. DADA2 was run as described in DADA2 Pipeline https://benjjneb.github.io/dada2/tutorial.html using the default parameters. In order to improve the overall quality of the sequences, the reads were filtered and trimmed using the filterAndTrim function implemented in DADA2. To remove low‐quality bases at the end of the reads, the truncLen option was set to 280 and 220 for the forward and reverse fastq files, respectively. Moreover, to remove the adapter sequences at the 5′ end the trimLeft option was set to 17 and 21 (forward and reverse reads, respectively). The taxonomic assignment was performed using the naïve Bayesian classifier method implemented in DADA2 using as reference the SILVA^33^ database. A phylogenetic tree of the ASVs was obtained using the function AlignSeq implemented in DEPHER^34^ package to create the multiple sequence alignment and the R library phargon to create the final tree.

In order to remove artifact and very lowly abundant ASVs, we filtered all the ASVs that were not assigned to a phylum and that have an abundance lower than 0.005 and present in less than two samples.

### 2.6 Microbial community complexity and diversity analysis

The α‐diversity measures the complexity of a community within a sample. Several α‐diversity indexes have been calculated (Chao1, Shannon, Simpson, and Fisher), and Wilcoxon‐Mann‐Whitney test was used to compare the species richness between groups stratified by disease (healthy, CD, and UC) and treatment (controls, butyrate treated, and placebo treated).

A Permutational analysis of variance (PERMANOVA) was performed in order to explore the contribution of several variables to microbial composition (β‐diversity) such as the condition of disease or healthy population (IBD or healthy), the disease (CD or UC), the gender, or the treatment (butyrose or placebo). In a PERMANOVA, the different covariates of interest are tested sequentially into a regression model and through a permutational approach the analyses measure the contribution of each variable to explain the samples distribution. A low *P*‐value (*P* < .05) indicates that the considered variable significantly impacts on the microbial community.

### 2.7 Statistical data analysis

Clinical variables between treatment and control groups were tested using Mann‐Whitney test for numerical data and chi‐squared test for categorical data. When comparing clinical variables across times, Wilcoxon test was used. In Table S3, ASV abundances were compared using Wilcoxon‐Mann‐Whitney test. The *P*‐values were adjusted using FDR (FDR ≤ 0.1 was used as a significance cutoff).

Statistical analysis was performed on R (Version 3.4.4), and the following R packages were used to analyze microbiome data: phyloseq (version 1.24.0) to facilitate the import, storage, analysis, and graphical display of microbiome census data^35^; Vegan (version 2.4.2) for PERMANOVA. Data were preprocessed removing possible contaminants (mythocondrial and chloroplast sequences) and filtering too rare features. PERMANOVA was computed with andonis2 function of Vegan package and *betadisper* function of the same package for graphical output. For PERMANOVA, data were normalized through rarefaction in order to take into account the different sample sequencing depth. In order to have a qualitative information about most discriminant features in the dataset, we compute sparse partial least squares discriminant analysis with *plsda*, *tune.splsda*, and *splsda* functions of mixOmics (6.3.1) R package.^36^ For the latter, we follow default pipeline: data normalization with total sum scaling and adding a pseudo‐count value of 1 (to raw data) to avoid issues when computing centered log‐ratios. On top discriminant features for each comparison, a Wilcoxon‐Mann‐Whitney test is performed on relative abundances and the Benjamini‐Hochberg multiple testing correction procedure is applied. The *P*‐values reported in the text will refer to the adjusted *P*‐values.

## 3 RESULTS

Among 65 consecutive patients assessed for eligibility, three did not meet inclusion criteria, four declined to participate, and one did not provide fecal material. Fifty‐seven patients were randomized to receive either microencapsulated butyrate (BLM) or placebo (PBO; flow diagram has been illustrated in Figure S1). At the end of the study, data from forty‐nine patients (19 CD/30 UC, 36M/13F, mean age 51) were available and further analyzed. Eighteen healthy subjects (7M/11F), mean age 29, were also recruited. The demographic and clinical characteristics of enrolled IBD patients stratified according to treatment are depicted in Table 1. Demographic and clinical features did not differ between the two groups. As to the control group, healthy volunteers (HVs) were generally younger (mean age HVs 29 vs IBD 51, *P* = .0004).

**Table 1:** Patient baseline characteristics

	IBD all population	Treatment group	Placebo group	Adj.*P*
Male, n, %	36, 73.46	15, 71.4	21, 75	1
Median Age, years	51 (19‐73)	51(19‐69)	50(25‐73)	1
Median BMI	24.12(16.04‐30.02)	23.84	24.21	1
Type of disease, n, %	CD, 19, 38.77	7	12	1
Montreal classification UC, n, %				
E1	2, 6.6	1	1	1
E2	13, 43.3	6	7	
E3	15, 50	7	8	
CD behavior, n, %				
B1	16, 84.2	4	12	.08
B2	3, 15.7	3	—	
B3	0	—	—	
Location, n, %				
L1	5, 26.3	3	2	.48
L2	5, 26.3	—	5	
L3	9, 47.3	4	5	
Endoscopic score				
Mayo score, n, %				
0	14, 46.6	7	7	1
1	8, 26.6	4	4	
2	5, 16.6	3	2	
3	3, 10	—	3	
SES‐CD, n, %				
0‐2	9, 47.36	3	6	1
3‐6	7, 36.8	3	4	
7‐15	3, 15.7	1	2	
>15	0	—	—	
Previous surgery n (CD‐UC), %	7, 31.5	6 (5‐1)	1 (1‐0)	.12
Smokers CD, UC	3, 2	2	3	1
Therapy				
Biologics n, %	20, 40.8	8	12	1
5‐ASA n, %	45, 91.8	20	25	1
Probiotics(ECN) n, %	4, 8.1	2	2	1
Steroids n, %	7, 14	1	6	.84
Immunosuppressant n, %	6, 12.2	3	3	1
PPI	7, 14	1	6	.84

Note

Baseline characteristics of CD (n = 19) an UC (n = 30) patients allocated on the butyrose(treatment) or placebo groups. The adj.*P*‐value was calculated as described on the statistical data analysis.

### 3.1 16S metagenomics analysis

**Figure 1:**
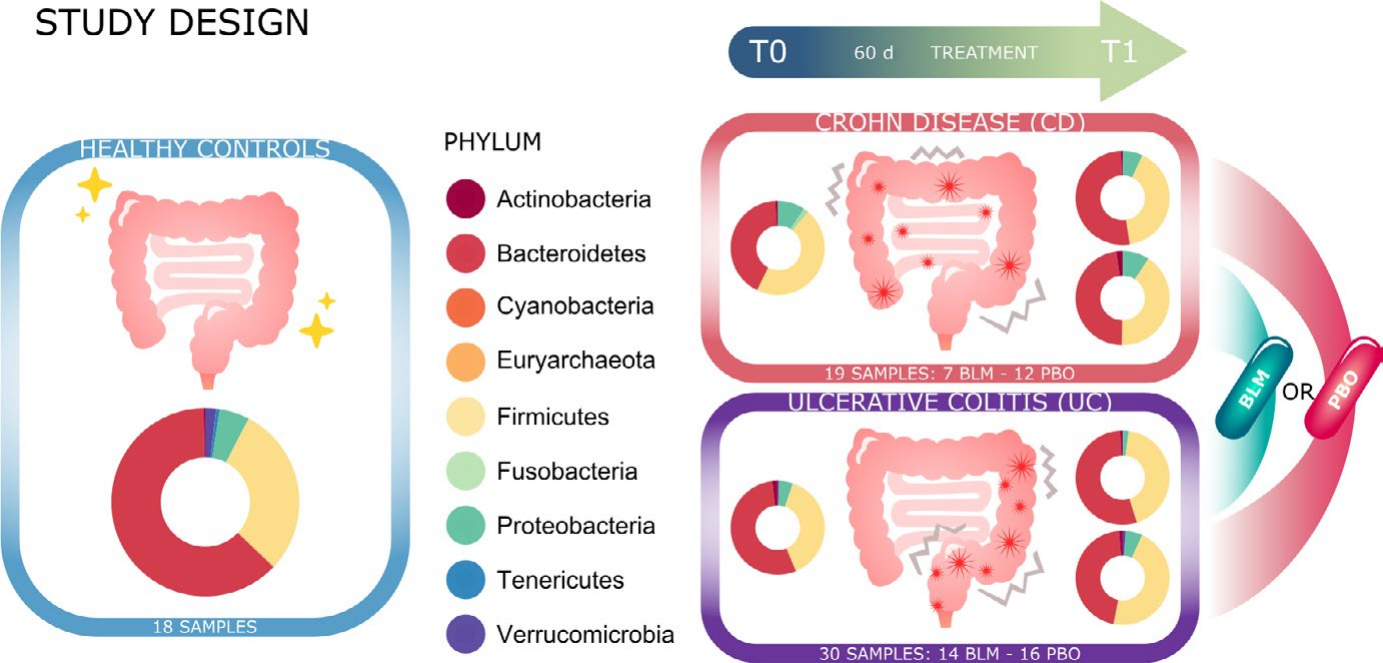
Graphic summary. Project study design: 18 healthy subjects and forty‐nine patients (21 on butyrose group and 28 on placebo group; 19 CD patients and 30 UC patients) were enrolled for this study. Pie charts show the microbial composition at phylum level in the different groups of samples

A total of 9.652.259 paired‐end sequences (an average of 83.209 reads per sample) with a read length of 300 bp were obtained. After reads, quality check, denoizing, and chimera filtering (see material and methods for details), 2852 ASVs were found. Several filters based on taxonomic classification and ASV abundance were applied in order to remove ASV artifacts (see experimental procedures for more details). After this filtering step, a total of 927 different ASVs were obtained. The taxonomy classification allowed to identify 9 phyla, 18 classes (927 ASVs), 23 orders (927 ASVs), 33 families (915 ASVs), 125 genera (808 ASVs), and 98 species (158 ASVs). The comparison of rarefaction curves (Figure S2) as a function of sampling depth was performed. Results showed that all curves were close to saturation, indicating the richness of samples was fully observed/sequenced^37^ (Figure 1 or Graphic summary).

### 3.2 Treatment effects on α‐diversity: the intra‐individual diversity

**Figure 2:**
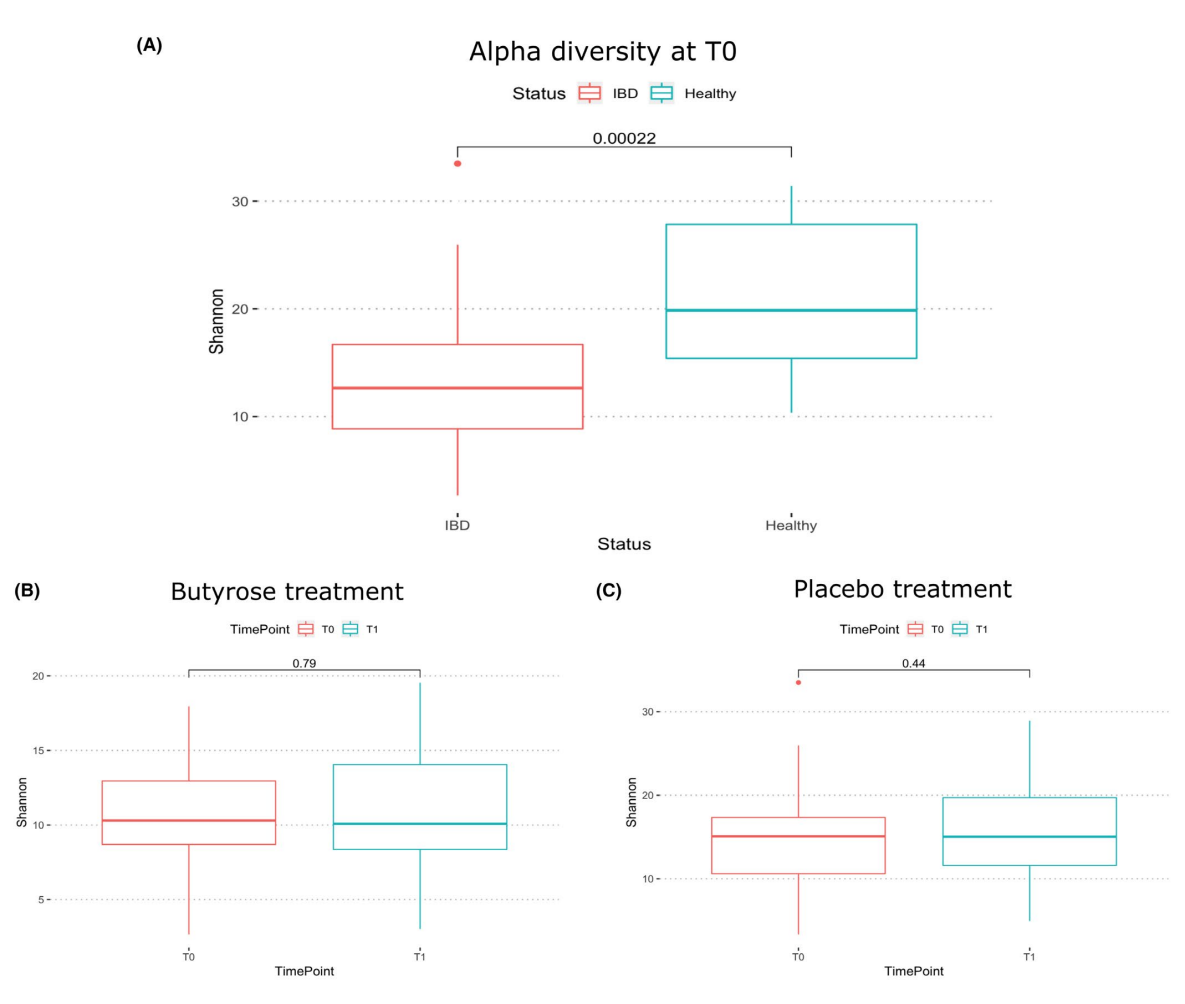
Box‐plot comparison of the alfa diversity calculated using Fisher metric between IBD and healthy group (A), timepoint T0 and T1 within the BLM group (B), and PBO group (C). Analysis performed with other distances confirms the same results (data not shown)

At baseline (T0), we observed a significant lower microbiota richness (*P* < .001) in the IBD patients compared with HVs (Figure 2, panel A). After treatment with PBO or BLM (T1), we did not observe any significant difference in terms of richness (Figure 2, panels B and C). While this was expected for the placebo, these results suggest that the short‐term butyrate treatment did not have an effect on increasing the complexity of the microbial community.

### 3.3 Treatment effects on β‐diversity: the inter‐individual diversity

**Figure 3:**
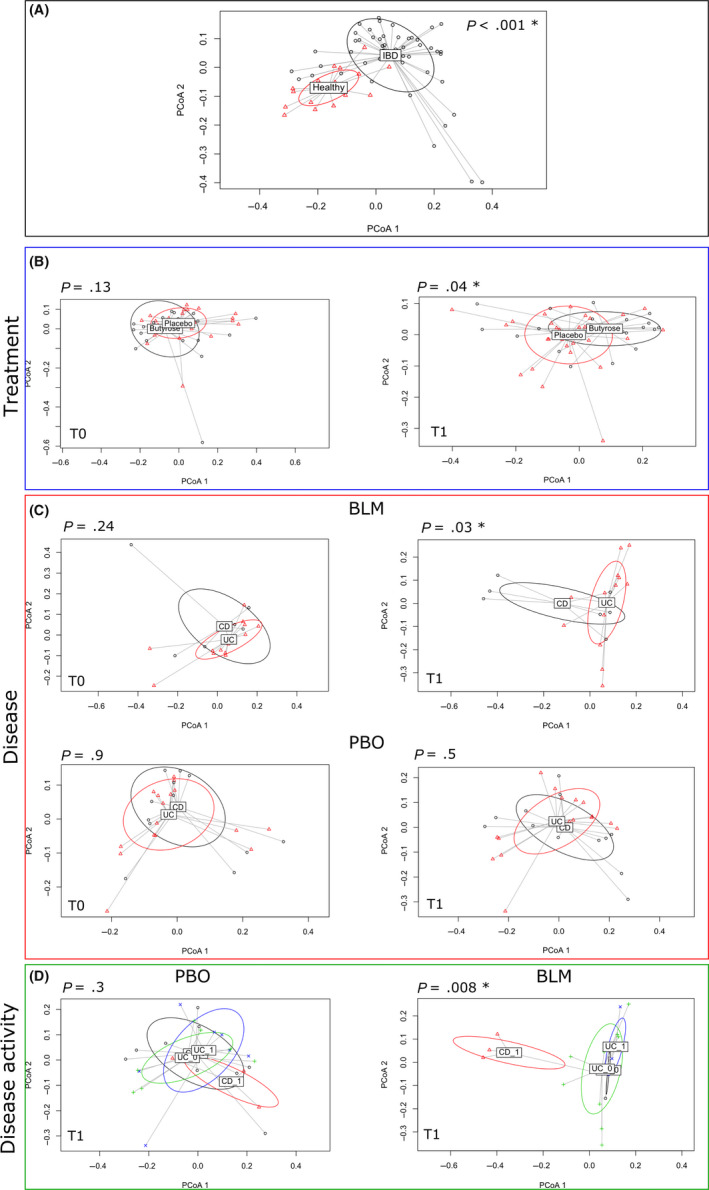
PERMANOVA tests if samples can be significantly separated accordingly to different variables (eg, treatment or type of disease). The figure shows the principal coordinate analysis considering the samples grouped according to (A) healthy and IBD status, (B) treatment (placebo and butyrose) at T0 = baseline and T1 = post‐treatment, (C) disease (UC and CD) undergoing BLM or PBO treatment at T0 and T1 timepoint, and (D) disease activity (1 indicates an active disease, while 0 a non‐active disease). The *P*‐value derived from the PERMANOVA test is reported for each comparison, and significant *P*‐value (<0.05) is indicated with a star

Firstly, we performed a PERMANOVA using the phylogenetic unweighted UniFrac distance on HV and IBD groups before the treatment. As shown in the principal coordinates analysis (PCoA) plot in Figure 3, panel A, HVs were clearly segregated from the IBD patients (*P* < .001), because of a different bacterial composition between the two groups. Then, we focused the analysis on the IBD groups: PERMANOVA showed that after treatment a significant difference (*P* = .045) occurred between BLM and PBO groups (Figure 3, panel B), whereas this difference was not significant at baseline (*P* = .13).

Finally, we further stratified the groups according to the type of disease. After the treatment (T1) on the BLM arm, we found a clear separation between CD and UC patients (Figure 3, panel C, *P* = .030), also considering disease activity (*P* = .00835, Figure 3, panel D). Beforehand we verified that at baseline, there were no differences between the CD and UC patients allocated on the BLM arm and PBO arm to confirm the homogeneity of the groups before the treatment (Figure 3, Panel C).

### 3.4 Microbiota composition of IBD patients and HVs before and after treatment

**Figure 4:**
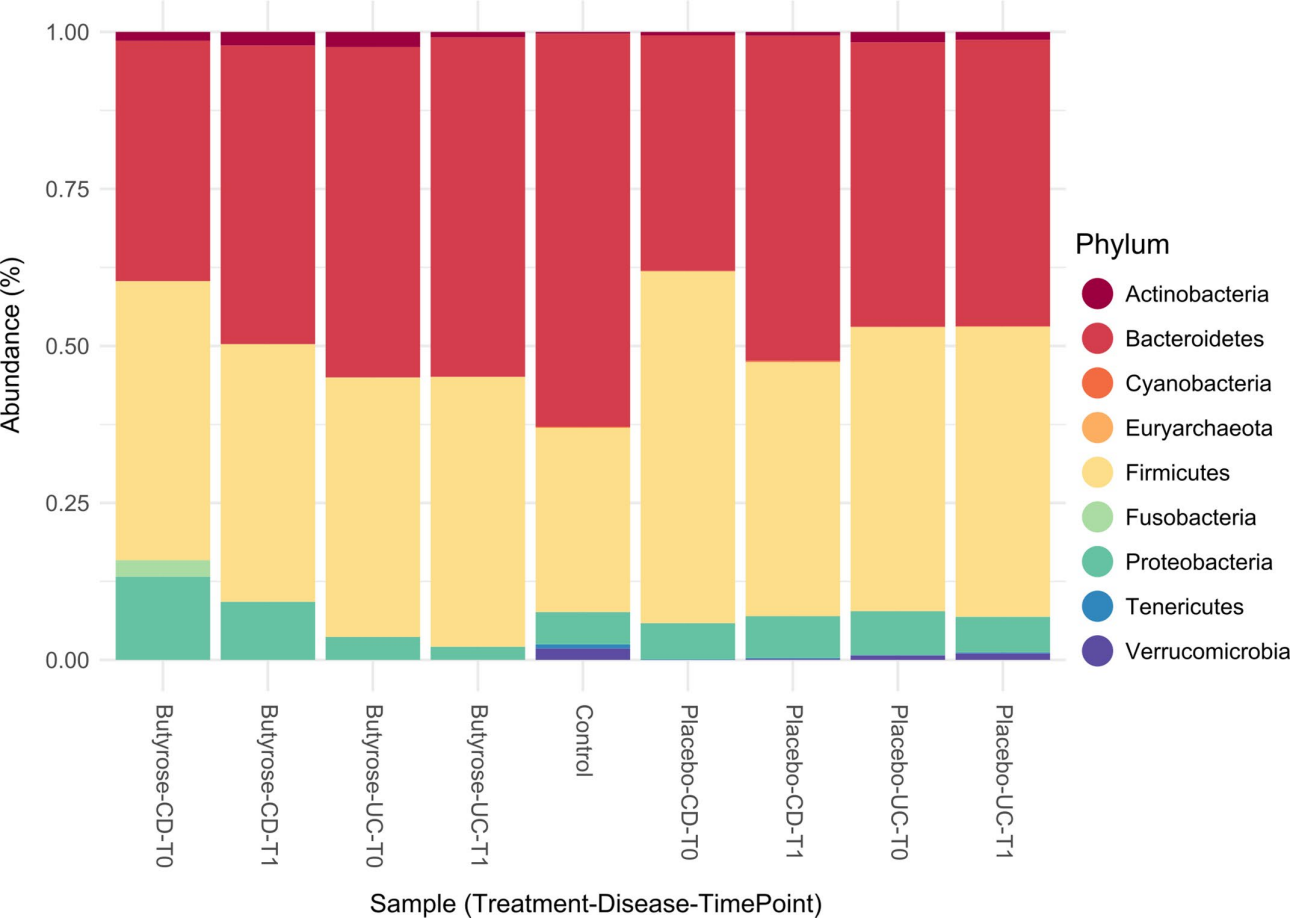
Barplot of the microbial composition at phylum level: average ASV abundance percentages of the samples stratified by treatment (butyrose, placebo, or control), disease (CD, UC, or control), and timepoint (T0 = baseline or T1 = post‐treatment)

Phylum microbiota profile is represented in Figure 4 and Table S1. At baseline, HVs showed a different microbiota composition compared with IBD patients, although the differences between HVs and patients in PBO arm were less pronounced (Table S1).

In particular, HVs showed a higher abundance of *Verrucomicrobia* (*P* = .0194) and possibly *Tenericutes* (*P* = .0733) compared with CD patients in the BLM group. *Fusobacteria* (*P* = .0733) appeared more abundant in CD patients in the BLM group compared with HVs. Moreover, HVs differed higher from UC patients in the BLM group with respect to *Verrucomicrobia* (*P* = .0004) and *Tenericutes* abundance (*P* = .0059). As to the PBO group, HVs showed a higher abundance of *Verrucomicrobia* (*P* = .0011) compared with CD patients, whereas both *Verrucomicrobia* (*P* = .0096) and *Tenericutes* (*P* = .0583) were more abundant in UC patients compared with HVs. In contrast, *Actinobacteria* were more abundant (*P* = .0814) in UC patients compared with HVs.

After treatment, both BLM and PBO groups showed almost the same differences in terms of phylum composition compared with HVs. Indeed, only a reduction in *Proteobacteria* in UC (*P* = .0428) compared with HVs was observed. However, to realistically assess differences in microbiota composition before and after treatment, we performed a deeper taxonomical level analyses (Sparse Partial Least Squares Discriminat Analysis [SPLS‐DA]), see below under “Differences in Microbiota Composition between IBD and Controls” (Figure 4).

### 3.5 Differences in microbiota composition between IBD and controls

**Figure 5:**
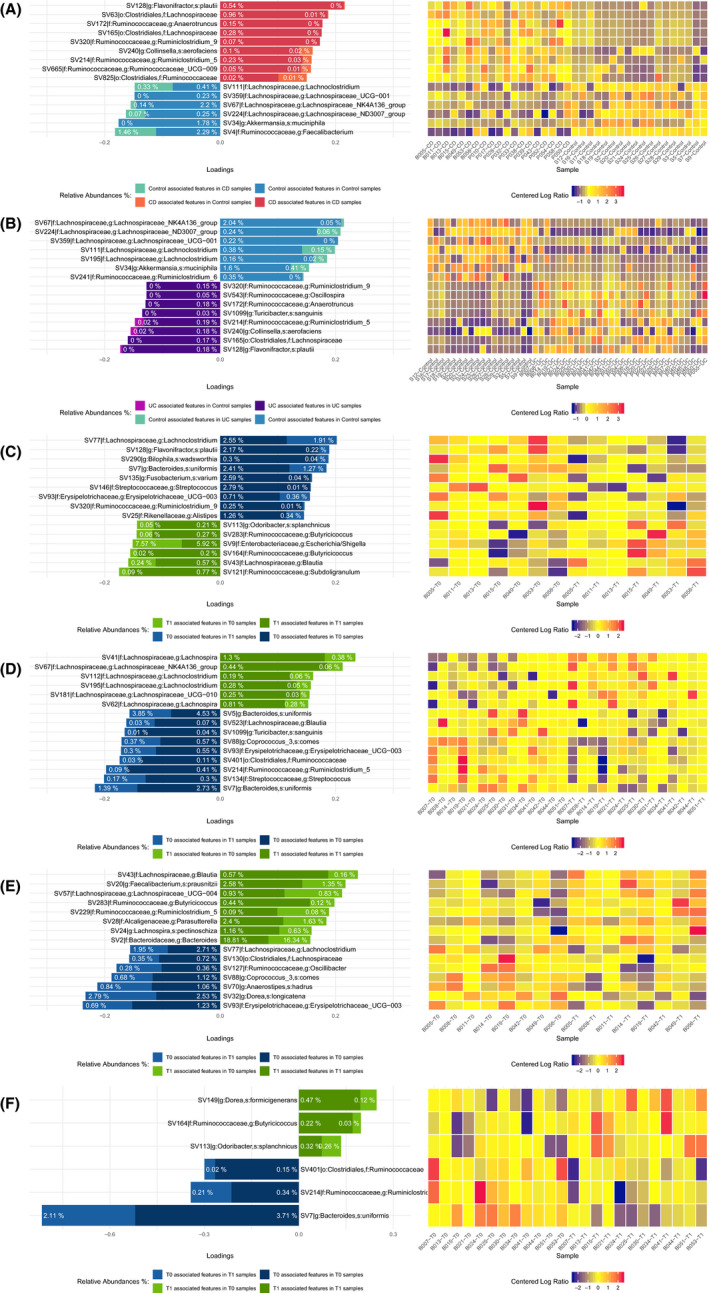
sPLS‐DA analysis identified a subset of discriminant ASVs: for each ASV, a loading value that represents the discriminant power of that ASV in explaining differences between 2 examined conditions. The higher the absolute value, the bigger is the discriminative power. The loading value plots display the top 15 (panels A‐E) and top 6 (panel F) discriminant ASVs for each comparison. Percentages shown in the bars represent the mean relative abundances of each ASV in the considered conditions. The dark color and a light color of each bar represent the average relative abundance (in percentage) of an ASV in the considered comparison

With the Sparse Partial Least Squares Discriminat Analysis (SPLS‐DA), it is possible to discriminate ASVs that best characterize each group as shown in Figure 5 and Table S2. The SPLS‐DA analysis identified several differences in the microbiota composition between HVs and IBD patients. In CD patients (Figure 5, panel A and Table S2A), a reduction (*P* < .01) in *Feacalibacterium genus*, *Akkermansia muciniphila*, and some *Lachnospiraceae family* was observed compared with HVs. Furthermore, we found a significant increase (*P* < .01) in *Flavonifractor plautii* and *Collinsella aerofaciens* besides some *Lachnospiraceae* ssp Among UC patients (Figure 5, panel B), we observed a strong reduction (*P* < .001) in *Lachnospiraceae* family, *Ruminoclostridium_6* and *A muciniphila*, and an enrichment (*P* < .01) of several *Ruminococcaceae*, such as *Oscillospira*,* Rumininiclostridium*, and *Anaerotruncus* genus compared with HVs. We also observed an increase (*P* < .01) in *F plautii*, and *C aerofaciens* as already noted on CD patients, as well as *Turicibacter sanguinis*.

After BLM treatment, in CD patients we did not find any significant change in microbiota composition (Figure 5, panel C and Table S2C). However, we observed a mild enrichment of butyrate producer genus *Butyricicoccus* and a reduction in *Lachnoclostridium*, *F plautii*, *Bilophila wadsworthia*, and *Erysipelotrichaceae_UCG.003*. After BLM treatment, in UC patients (Figure 5, panel D and Table S2D), we observed an increase in *Lachnospiraceae* family (SCFA producers; *P* ≅ 0.1) and a reduction in *Bacteroides uniformis*,* Blautia*,* T sanguinis*,* Erysipelotrichaceae_UCG.003*, and *Ruminococcaceae* family (*P* ≅ .1).

The bacterial composition changes appeared influenced by disease activity (Figure 5, panel E‐F and Table S2E‐F). In case of disease remission, we found a significant increase in *Dorea formicigenerans* and *Butyricicoccus* (*P* < .073), while a decrease in *Ruminococcaceae* family and *B uniformis* was noted (*P* < .073). In case of clinical activity, no significant changes were found. However, we observed a mild increase in the *Blautia* genus,* F prausnitzii*, and *Lachnospira pectinoschiza* and a mild decrease in *Erysipelotrichaceae* genus and *Anaerostipes hardus*.

### 3.6 Treatment effects on clinical activity, fecal calprotectin, and IBDQ

We did not observe any effect on clinical activity between the two groups of treated patients, both in terms of pMS (*P* = .06) and in terms of HBI (*P* = .8), although in UC patients the pMS value tended to be significant (as shown in Table S3). Similar results were obtained when we evaluated the FC levels (Table S4 and Table 2). Subjective improvement in QoL based on IBDQ was observed in the BLM treatment (*P* = .0184) and less in the PBO (*P* = .156) group, although the greatest effect was observed in UC patients treated with BLM (*P* = .0284; Table 3).

**Table 2:** The decrease in fecal calprotectin levels for CD ( above 250 µg/g) and UC (above 150 µg/g)

	CD (≥250 µg/g)		*P*	UC (≥150 µg/g)		*P*
	B (%)	PBO (%)		B (%)	PBO (%)	
Reduction of 30% of FC index	67	37.5	.8	57.1	55.5	.9

Abbreviations: B, treatment with butyrose; PBO, no treatment.

**Table 3:** The improvement in QoL based on IBDQ

Treatment	Disease	No of. patients	IBDQ (T0/T1) median	Adj.*P*
B	CD T0 vs CD T1	7	173/191	1
B	UC TO vs UC T1	14	170/193.5	.0284*
PBO	CD T0 vs CD T1	12	174.5/179.5	.2364
PBO	UC T0 vs UC T1	16	188/188	.5432

Abbreviations: B, treatment with butyrose; PBO, no treatment.

* High significance.

## 4 DISCUSSION

Short‐chain fatty acids (SCFAs, mainly acetate, propionate, and butyrate) are produced by anaerobic bacterial fermentation from dietary fibers within the lumen of the mammalian colon.^3^ They play important roles in colonic homeostasis.^38^ It has been hypothesized that the influence of SCFA on microbiota composition may have a relevant impact on IBD and its disease activity.^39^ Herein, for the first time we performed a double‐blind, randomized, controlled, pilot study aimed to analyze the effect of an oral butyrate treatment on fecal microbiota composition in patients with IBD. Evaluating 49 subjects, we found that butyrate could alter the gut microbiota of IBD patients by increasing the bacteria able to produce SCFA in both UC and CD patients. Butyrogenic colonic bacteria particularly become predominant in CD patients. Moreover, butyrate administration determined an improvement of QoL in UC.

At baseline, the microbiota composition of HVs differed from that of IBD patients. After treatment, both BLM and PBO groups showed almost the same differences in terms of phylum composition compared with HVs and therefore no effect at phylum level. Thus, we showed the persistence of a low complexity of the microbial community (α‐diversity) before and after treatment (for both BLM and PBO groups), suggesting that the short‐term treatment (8 weeks) did not increase the α‐diversity. Regarding phylum alterations, this can be expected, as butyrate being a safe bacterial product does not show the drastic effects expected to see when modulating gut microbiota with fecal microbiota transfer or antibiotics.^40^

As described in the literature,^1^, ^11^, ^40^, ^41^, ^42^ we confirmed using PERMANOVA the evidence of a significant difference (*P* < .001) between the microbiota of HVs and IBD patients, documenting dysbiosis in IBD subjects.^41^ Moreover, the PERMANOVA permitted us to observe at the end of follow‐up, a significant effect of BLM treatment compared with PBO in modifying the composition of the gut microbiota. The same difference was observed by stratifying the groups according to the type of disease (CD vs UC), suggesting a significantly different treatment effect dependent on the type of disease (*P* = .03) and disease activity (*P* = .00835). The latter differences were not found in the PBO group, further corroborating the biologic effect of BLM administration.

With discriminant analysis, we evaluated the specific bacteria characterizing the gut microbiota on treated patients at baseline and after treatment. At baseline, CD patients presented a reduction in *F prausnitzii*,* A muciniphila*, and the *Lachnospiraceae family* as compared to healthy controls, bacteria that are considered as a dysbiosis‐marker in IBD patients.^42^, ^43^ Moreover, CD patients showed an increase in the *Ruminococcaceae* family and *F plautii*,* C aerofaciens*, associated with increased risk of developing colon cancer because of the extensive degradation of flavonoids by gut microflora (*F plautii*) ^44^and a marker of a low dietary fiber intake.^45^ In UC patients, we highlighted a reduction in *Lachnospiraceae* family and an enrichment of several *Ruminococcaceae*, such as *Oscillospira*,* Rumininiclostridium*, and *Anaerotruncus* genus compared with the HVs. We also observed an increase in *F plautii* and *C aerofaciens* as already noted in CD patients, as well as the *T sanguinis* associated with impaired intestinal permeability.^46^

After treatment, in CD patients, we observed a mild enrichment of butyrate producer genus *Butyricicoccus*, while in UC patients, we found an increase in generic SCFA producers (*Lachnospiraceae* spp.). These results support the potential effect of butyrate in increasing the butyrogenic producers, which anyway requires further confirmatory data including more patients. The former finding confirmed the data of Louis et al,^9^ who showed an overall reduced genetic capacity from the gut microbiome to synthesize butyrate in CD patients. Furthermore, our study showed for the first time that an oral microencapsulated butyrate administration seems to promote the growth of bacteria able to increase the production of butyrate. We speculate that this phenomenon was due to the effect of administered butyrate, allowing the eubiotic restoration (eg, Clostridia) at the level of the mucosal microbiota involved in the maintenance of intestinal immune homeostasis, as suggested by Spees AM.^47^ On the other hand, in UC patients, where the condition of reduced genetic capacity for butyrate synthesis was not described,^2^ the BLM administration stimulated the growth of generic although useful SCFA producers.

Recently, fecal microbiota profile has been shown as a biomarker for disease activity in CD,^48^ and herein, we confirmed these evidence albeit with a weak clinical evidence. Indeed, according to baseline clinical activity, we found a mild decrease in butyrate producer genera (*Blautia* and *Faecalibacterium*) and a mild increase in *Erysipelotrichaceae_UCG.003* (SV93), which resulted to be partially reduced after BLM treatment. *Erysipelotrichaceae* genus has been found highly immunogenic and positively correlated with tumor necrosis factor alpha.^49^ Thus, the reduction in these bacteria induced by BLM may have had clinical implication (ie, improvement of quality of life). At baseline, in disease remission patients we found an increased prevalence of bacteria more often associated with healthy state, like *B uniformis*.^50^ Moreover, we observed that after active treatment, bacteria (*D formicigenerans*,* Butyricicoccus*) associated with a healthy gut microbiome were more prevalent.

As to the clinical activity, we did not find any difference in terms of outcome between the two groups. The medical literature is rich in data, suggesting that butyrate exerts multiple favorable effects such as the prevention and inhibition of colonic carcinogenesis, the improvement of inflammation, oxidative status, epithelial defense barrier, and the modulation of visceral sensitivity and intestinal motility.^51^ However, subjective QoL improvement based on IBDQ was significantly observed either both in the treatment (*P* = .0046) and in the PBO (*P* = .039) groups, although a greater effect was found after BLM treatment (*P* = ns). Similar results were reported by Banasiewicz et al where a BLM supplemental therapy significantly decreased bowel symptoms after 4 weeks of treatment.^52^

Some limitations of the current study have to be acknowledged. First, we failed to observe significant changes in terms of disease activity after treatment. This could be due to the small sample size and the fact that the majority of our patients were in remission. For the same reasons, we had to include in the same study population both patients with UC and CD, with different disease activities and treatments, and this could also be seen as a limitation. A similar consideration can be done for the short‐term treatment administration. However, this latter data were part of the secondary aim of the study, whereas our primary aim was to observe the effect of butyrate on microbiota composition. For this reason and for their potential clinical implications, we decided to include them and speculate on their involvement in IBD management. Second, we did not perform a cross‐over study to further validate our findings. Finally, our HVs differed compared with the patients in terms of mean age and gender, and this may have affected our results. However, it is relevant to note that the microbiota characteristics of our HVs were similar to those frequently described in medical literature in older subjects, and therefore, these differences could be less relevant for our results.^41^

In conclusion, in this pilot study, we evaluated the effect of a sodium butyrate microencapsulated oral formulation (*Butyrose^R^ Lsc Microcaps*) on the gut microbiota of IBD patients. Recently, it was highlighted that the lack of butyrate may alter the gut homeostasis, increasing oxygen concentration in the lumen and therefore reducing the concentration of butyrate‐producing bacteria.^53^ Our study emphasizes how the oral supplementation of exogenous butyrate can modulate the gut bacteria, stimulating the growth of butyrogenic and SCFA genera which in turn may produce more endogenous butyrate for intestinal wellness. Further studies are necessary to evaluate the clinical impact of oral administration of exogenous butyrate effect on clinical activity and mucosal healing in IBD patients.

## CONFLICT OF INTEREST

The authors report no conflict of interest.

## AUTHOR CONTRIBUTIONS

SF, AB, and EVS involved in study concept and design; SF, NV, MC, BP, and CR analyzed and interpreted the data; SF, NV, DP, MC, CR, and EV. S. drafted the manuscript, critically revised the manuscript for important intellectual content. DP, GL, CM, R.D’I., and GC.S. performed acquisition of data and critical revision of the manuscript. NV, CR, and MC carried out statistical analysis. All authors read and approved the final manuscript.

## Supporting information

Supplementary Material
