# Supplementary material for: Microbiota changes induced by microencapsulated sodium butyrate in patients with inflammatory bowel disease
Source: Neurogastroenterol Motil. 2020 May 31;32(10):e13914. doi: 10.1111/nmo.13914 (PMC7583468; doi:10.1111/nmo.13914)
Supplement: Supplementary file 1 — Supplementary Material [file NMO-32-e13914-s001.docx]

**Supporting information**

**S 1:** The following diagram represents the patient enrollment procedure used in the study as indicated.

Analysed (n=21)
♦ Excluded from microbiota analysis : (n=1 ) no reaction in Polymerase chain reaction

Lost to follow-up (not traced,n=2 )

Discontinued intervention (1:uncompliance;2: take antibiotics; 3: take probiotics) (n=3)

1 )

## Follow-Up

## Analysis

Analysed (n=28)
♦ Excluded from analysis (n= 0)

Lost to follow-up (not traced, n=1)

Discontinued intervention (n=0 )

## Enrollment

Allocated to Butyrose (n=28)

♦ Received allocated intervention (n= 27 )

♦ Did not receive allocated intervention (n=1 hospitalization )

## Allocation

Allocated to Placebo (n=29)

♦ Received allocated intervention (n=29)

♦ Did not receive allocated intervention (n= 0)

Randomized (n= 57)

Excluded (n=8)

♦ Not meeting inclusion criteria (n=3)

♦ Declined to participate (n=4)

♦ Other reasons (n= 1)

Assessed for eligibility (n=65)

**Flow Diagram**

**S 2 Rarefaction curve**

**
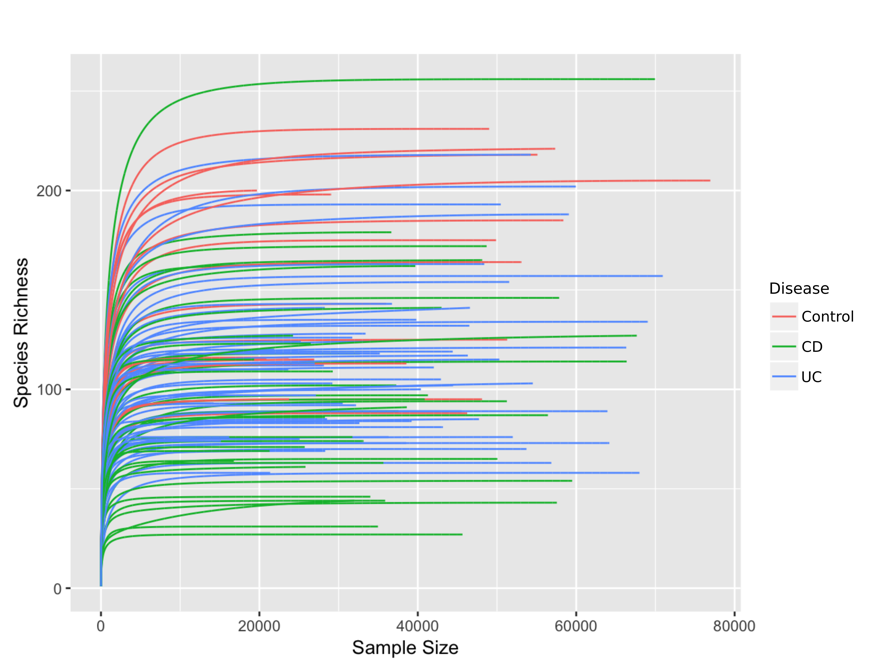
**

Rarefaction curve of all the samples, stratified by disease/control. The plot shows that all the samples reach species richness saturation, indicating an adequate sequencing depth.

| **Table S 3 A) ADJUSTED P-VALUES FOR PHYLUM LEVEL COMPOSITION TESTS** | | | | | | | | | |
| --- | --- | --- | --- | --- | --- | --- | --- | --- | --- |
| Comparison | Actinobacteria | Bacteroidetes | Cyanobacteria | Euryarchaeota | Firmicutes | Fusobacteria | Proteobacteria | Tenericutes | Verrucomicrobia |
| ButyroseCDT0_vs_ButyroseCDT1 | 1,0000 | 1,0000 | 1,0000 | 1,0000 | 1,0000 | 1,0000 | 1,0000 | 1,0000 | 1,0000 |
| ButyroseCDT0_vs_ControlControlT0 | 0,4735 | 0,8382 | 0,3612 | 0,3172 | 0,9764 | 0,0733 | 0,8382 | 0,0733 | 0,0194 |
| ButyroseCDT1_vs_ControlControlT0 | 0,9407 | 0,9764 | 0,3612 | 0,3172 | 0,9407 | 0,0733 | 0,9407 | 0,0733 | 0,0194 |
| ButyroseUCT0_vs_ButyroseUCT1 | 1,0000 | 1,0000 | 1,0000 | 1,0000 | 1,0000 | 1,0000 | 1,0000 | 1,0000 | 1,0000 |
| ButyroseUCT0_vs_ControlControlT0 | 0,2205 | 0,4341 | 0,2073 | 0,2205 | 0,9745 | 1,0000 | 0,2205 | 0,0059 | 0,0004 |
| ButyroseUCT1_vs_ControlControlT0 | 0,3933 | 0,2252 | 0,1555 | 0,3933 | 0,7499 | 1,0000 | 0,0428 | 0,0262 | 0,0015 |
| PlaceboCDT0_vs_PlaceboCDT1 | 1,0000 | 1,0000 | 1,0000 | 1,0000 | 1,0000 | 1,0000 | 1,0000 | 1,0000 | 1,0000 |
| PlaceboCDT0_vs_ControlControlT0 | 0,8595 | 0,8595 | 0,8595 | 0,8595 | 0,8595 | 1,0000 | 1,0000 | 0,2655 | 0,0011 |
| PlaceboCDT1_vs_ControlControlT0 | 0,8314 | 0,8314 | 0,8314 | 0,8314 | 0,8314 | 1,0000 | 0,8314 | 0,8202 | 0,0259 |
| PlaceboUCT0_vs_PlaceboUCT1 | 1,0000 | 1,0000 | 1,0000 | 1,0000 | 1,0000 | 1,0000 | 1,0000 | 1,0000 | 0,8979 |
| PlaceboUCT0_vs_ControlControlT0 | 0,0814 | 0,6227 | 0,3827 | 0,6227 | 0,8107 | 1,0000 | 0,6227 | 0,0583 | 0,0096 |
| PlaceboUCT1_vs_ControlControlT0 | 0,1450 | 1,0000 | 0,4235 | 0,8414 | 0,6026 | 0,5696 | 0,6026 | 0,1525 | 0,0483 |
| Adjusted p.value < 0.05 |  |  |  |  |  |  |  |  |  |
| Adjusted p.value < 0.1 |  |  |  |  |  |  |  |  |  |
| **B) MEAN DIFFERENCE BETWEEN MEAN PROPORTIONS (%) OF GROUP A AND B** | | | | | | | | | |
| Group A vs Group B | Actinobacteria | Bacteroidetes | Cyanobacteria | Euryarchaeota | Firmicutes | Fusobacteria | Proteobacteria | Tenericutes | Verrucomicrobia |
| ButyroseCDT0_vs_ButyroseCDT1 | 0,1864 | -7,1041 | 0,0000 | 0,0000 | 2,2684 | 2,2794 | 2,3699 | 0,0000 | 0,0000 |
| ButyroseCDT0_vs_ControlControlT0 | 1,4018 | -7,4209 | -0,2254 | -0,0603 | -0,5906 | 2,3016 | 6,6388 | -0,5060 | -1,5389 |
| ButyroseCDT1_vs_ControlControlT0 | 1,2154 | -0,3168 | -0,2254 | -0,0603 | -2,8590 | 0,0221 | 4,2689 | -0,5060 | -1,5389 |
| ButyroseUCT0_vs_ButyroseUCT1 | 1,5116 | -3,3629 | 0,0000 | -0,0018 | 1,2191 | 0,0000 | 0,6801 | -0,0166 | -0,0295 |
| ButyroseUCT0_vs_ControlControlT0 | 2,0533 | 2,6750 | -0,2254 | -0,0520 | -0,2889 | 0,0000 | -2,1172 | -0,5060 | -1,5389 |
| ButyroseUCT1_vs_ControlControlT0 | 0,5417 | 6,0379 | -0,2254 | -0,0501 | -1,5079 | 0,0000 | -2,7974 | -0,4894 | -1,5094 |
| PlaceboCDT0_vs_PlaceboCDT1 | -0,1507 | -0,7741 | -0,1432 | -0,0366 | 0,9141 | 0,0000 | 0,4662 | -0,0797 | -0,1960 |
| PlaceboCDT0_vs_ControlControlT0 | 0,0646 | 2,6637 | -0,2060 | 0,0291 | -1,2414 | 0,0000 | 0,6209 | -0,3919 | -1,5389 |
| PlaceboCDT1_vs_ControlControlT0 | 0,2153 | 3,4378 | -0,0628 | 0,0657 | -2,1555 | 0,0000 | 0,1547 | -0,3122 | -1,3430 |
| PlaceboUCT0_vs_PlaceboUCT1 | -0,1179 | 3,1710 | -0,0226 | -0,0606 | -2,6300 | -0,0484 | -0,1437 | -0,0654 | -0,0824 |
| PlaceboUCT0_vs_ControlControlT0 | 0,7162 | 0,9442 | -0,2213 | 0,0503 | 0,5620 | 0,0000 | -0,7462 | -0,4600 | -0,8453 |
| PlaceboUCT1_vs_ControlControlT0 | 0,8341 | -2,2268 | -0,1987 | 0,1109 | 3,1920 | 0,0484 | -0,6025 | -0,3946 | -0,7629 |
| Phyla more abundant in group B |  |  |  |  |  |  |  |  |  |
| Phyla more abundant in group A |  |  |  |  |  |  |  |  |  |
|  |  |  |  |  |  |  |  |  |  |

Adjusted p-value table (A) and mean differences table (B) of the comparisons between average ASVs abundance percentages at phylum levelof the samples stratified by Treatment (Butyrose, Placebo or Control), Disease (CD, UC or Control) and Timepoint (T0 or T1). Wilcoxon rank sum test and Mann-Whitney U test were applied for paired and unpaired data respectively.

**Table S 4**

| **TEST OF TOP DISCRIMINANT ASVs FOR EACH COMPARISON OF FIGURE 4** | | | | | | | | | | | | |
| --- | --- | --- | --- | --- | --- | --- | --- | --- | --- | --- | --- | --- |
| 1. **Controls vs CD (T0)** | | | | | | |  | **C) CD T0 vs T1 (Treated)** | | | | |
| SV | Proportion in Controls % | Proportion in CD % | | p-value | | Adjusted p-value |  | SV | Proportion in T0 % | Proportion in T1 % | p-value | Adjusted p-value |
| SV128.g.Flavonifractor.s.plautii | 0,0038 | 0,5407 | | 0,0004 | | 0,0011 |  | SV77.f.Lachnospiraceae.g.Lachnoclostridium | 2,5462 | 1,9077 | 0,0502 | 0,2264 |
| SV63.o.Clostridiales.f.Lachnospiraceae | 0,0066 | 0,9603 | | 0,0007 | | 0,0017 |  | SV128.g.Flavonifractor.s.plautii | 2,1707 | 0,2216 | 0,0907 | 0,2264 |
| SV172.f.Ruminococcaceae.g.Anaerotruncus | 0,0000 | 0,1475 | | 0,0057 | | 0,0085 |  | SV290.g.Bilophila.s.wadsworthia | 0,2955 | 0,0357 | 0,0907 | 0,2264 |
| SV165.o.Clostridiales.f.Lachnospiraceae | 0,0000 | 0,2814 | | 0,0057 | | 0,0085 |  | SV7.g.Bacteroides.s.uniformis | 2,4075 | 1,2727 | 0,1855 | 0,2264 |
| SV320.f.Ruminococcaceae.g.Ruminiclostridium_9 | 0,0000 | 0,0702 | | 0,0114 | | 0,0156 |  | SV135.g.Fusobacterium.s.varium | 2,5908 | 0,0379 | 0,1855 | 0,2264 |
| SV240.g.Collinsella.s.aerofaciens | 0,0163 | 0,0977 | | 0,0237 | | 0,0273 |  | SV146.f.Streptococcaceae.g.Streptococcus | 2,7897 | 0,0106 | 0,1855 | 0,2264 |
| SV214.f.Ruminococcaceae.g.Ruminiclostridium_5 | 0,0252 | 0,2334 | | 0,0232 | | 0,0273 |  | SV93.f.Erysipelotrichaceae.g.Erysipelotrichaceae_UCG.003 | 0,7144 | 0,3616 | 0,0907 | 0,2264 |
| SV665.f.Ruminococcaceae.g.Ruminococcaceae_UCG.009 | 0,0059 | 0,0482 | | 0,1209 | | 0,1209 |  | SV320.f.Ruminococcaceae.g.Ruminiclostridium_9 | 0,2462 | 0,0059 | 0,1855 | 0,2264 |
| SV825.o.Clostridiales.f.Ruminococcaceae | 0,0092 | 0,0189 | | 0,0728 | | 0,0780 |  | SV25.f.Rikenellaceae.g.Alistipes | 1,2590 | 0,3355 | 0,1807 | 0,2264 |
| SV111.f.Lachnospiraceae.g.Lachnoclostridium | 0,4141 | 0,3312 | | 0,0011 | | 0,0023 |  | SV113.g.Odoribacter.s.splanchnicus | 0,0520 | 0,2054 | 0,2113 | 0,2264 |
| SV359.f.Lachnospiraceae.g.Lachnospiraceae_UCG.001 | 0,2308 | 0,0000 | | 0,0001 | | 0,0009 |  | SV283.f.Ruminococcaceae.g.Butyricicoccus | 0,0621 | 0,2676 | 0,0907 | 0,2264 |
| SV67.f.Lachnospiraceae.g.Lachnospiraceae_NK4A136_group | 2,1993 | 0,1431 | | 0,0003 | | 0,0011 |  | SV9.f.Enterobacteriaceae.g.Escherichia.Shigella | 7,5659 | 5,9217 | 0,4170 | 0,4170 |
| SV224.f.Lachnospiraceae.g.Lachnospiraceae_ND3007_group | 0,2544 | 0,0653 | | 0,0003 | | 0,0011 |  | SV164.f.Ruminococcaceae.g.Butyricicoccus | 0,0173 | 0,2002 | 0,1855 | 0,2264 |
| SV34.g.Akkermansia.s.muciniphila | 1,7825 | 0,0000 | | 0,0000 | | 0,0007 |  | SV43.f.Lachnospiraceae.g.Blautia | 0,2395 | 0,5654 | 0,2113 | 0,2264 |
| SV4.f.Ruminococcaceae.g.Faecalibacterium | 2,2880 | 1,4645 | | 0,0016 | | 0,0030 |  | SV121.f.Ruminococcaceae.g.Subdoligranulum | 0,0886 | 0,7676 | 0,1855 | 0,2264 |
|  |  |  | |  | |  |  |  |  |  |  |  |
| 1. **Controls vs UC (T0)** | | | | | | |  | 1. **UC T0 vs T1 (Treated)** | | | | |
| SV | Proportion in Controls % | | Proportion in UC % | | p-value | Adjusted p-value |  | SV | Proportion in T0 % | Proportion in T1 % | p-value | Adjusted p-value |
| SV67.f.Lachnospiraceae.g.Lachnospiraceae_NK4A136_group | 2,0350 | | 0,0459 | | 0,0000 | 0,0000 |  | SV41.f.Lachnospiraceae.g.Lachnospira | 0,3792 | 1,3035 | 0,0264 | 0,1058 |
| SV224.f.Lachnospiraceae.g.Lachnospiraceae_ND3007_group | 0,2369 | | 0,0578 | | 0,0000 | 0,0001 |  | SV67.f.Lachnospiraceae.g.Lachnospiraceae_NK4A136_group | 0,0638 | 0,4412 | 0,0295 | 0,1058 |
| SV359.f.Lachnospiraceae.g.Lachnospiraceae_UCG.001 | 0,2160 | | 0,0000 | | 0,0000 | 0,0000 |  | SV112.f.Lachnospiraceae.g.Lachnoclostridium | 0,0613 | 0,1942 | 0,0295 | 0,1058 |
| SV111.f.Lachnospiraceae.g.Lachnoclostridium | 0,3824 | | 0,1519 | | 0,0000 | 0,0001 |  | SV195.f.Lachnospiraceae.g.Lachnoclostridium | 0,0532 | 0,2842 | 0,0502 | 0,1058 |
| SV195.f.Lachnospiraceae.g.Lachnoclostridium | 0,1632 | | 0,0235 | | 0,0000 | 0,0001 |  | SV181.f.Lachnospiraceae.g.Lachnospiraceae_UCG.010 | 0,0298 | 0,2485 | 0,0888 | 0,1058 |
| SV34.g.Akkermansia.s.muciniphila | 1,5984 | | 0,4112 | | 0,0001 | 0,0001 |  | SV62.f.Lachnospiraceae.g.Lachnospira | 0,2849 | 0,8066 | 0,0888 | 0,1058 |
| SV241.f.Ruminococcaceae.g.Ruminiclostridium_6 | 0,3505 | | 0,0046 | | 0,0001 | 0,0001 |  | SV5.g.Bacteroides.s.uniformis | 4,5332 | 3,8463 | 0,0917 | 0,1058 |
| SV320.f.Ruminococcaceae.g.Ruminiclostridium_9 | 0,0000 | | 0,1494 | | 0,0059 | 0,0068 |  | SV523.f.Lachnospiraceae.g.Blautia | 0,0744 | 0,0321 | 0,0295 | 0,1058 |
| SV543.f.Ruminococcaceae.g.Oscillospira | 0,0000 | | 0,0467 | | 0,0095 | 0,0095 |  | SV1099.g.Turicibacter.s.sanguinis | 0,0443 | 0,0058 | 0,0502 | 0,1058 |
| SV172.f.Ruminococcaceae.g.Anaerotruncus | 0,0000 | | 0,1845 | | 0,0036 | 0,0048 |  | SV88.g.Coprococcus_3.s.comes | 0,5742 | 0,3743 | 0,1473 | 0,1473 |
| SV1099.g.Turicibacter.s.sanguinis | 0,0000 | | 0,0252 | | 0,0095 | 0,0095 |  | SV93.f.Erysipelotrichaceae.g.Erysipelotrichaceae_UCG.003 | 0,5511 | 0,3005 | 0,0711 | 0,1058 |
| SV214.f.Ruminococcaceae.g.Ruminiclostridium_5 | 0,0219 | | 0,1874 | | 0,0039 | 0,0048 |  | SV401.o.Clostridiales.f.Ruminococcaceae | 0,1117 | 0,0299 | 0,0888 | 0,1058 |
| SV240.g.Collinsella.s.aerofaciens | 0,0152 | | 0,1756 | | 0,0008 | 0,0014 |  | SV214.f.Ruminococcaceae.g.Ruminiclostridium_5 | 0,4055 | 0,0905 | 0,0378 | 0,1058 |
| SV165.o.Clostridiales.f.Lachnospiraceae | 0,0000 | | 0,1671 | | 0,0013 | 0,0020 |  | SV134.f.Streptococcaceae.g.Streptococcus | 0,2973 | 0,1654 | 0,1024 | 0,1098 |
| SV128.g.Flavonifractor.s.plautii | 0,0037 | | 0,1775 | | 0,0003 | 0,0006 |  | SV7.g.Bacteroides.s.uniformis | 2,7319 | 1,3927 | 0,0711 | 0,1058 |
|  |  | |  | |  |  |  |  |  |  |  |  |
| **E) Treated IBD - with Disease Activity in T0 vs T1** | | | | | | |  | **F) Treated IBD - No Disease Activity in T0 vs T1** | | | | |
| SV | Proportion in T0 % | | Proportion in T1 % | | p-value | Adjusted p-value |  | SV | Proportion in T0 % | Proportion in T1 % | p-value | Adjusted p-value |
| SV43.f.Lachnospiraceae.g.Blautia | 0,1573 | | 0,5742 | | 0,0502 | 0,2156 |  | SV149.g.Dorea.s.formicigenerans | 0,1229 | 0,4670 | 0,0165 | 0,0729 |
| SV20.g.Faecalibacterium.s.prausnitzii | 1,3539 | | 2,5755 | | 0,0907 | 0,2156 |  | SV164.f.Ruminococcaceae.g.Butyricicoccus | 0,0348 | 0,2248 | 0,0295 | 0,0729 |
| SV57.f.Lachnospiraceae.g.Lachnospiraceae_UCG.004 | 0,8251 | | 0,9251 | | 0,6054 | 0,6054 |  | SV113.g.Odoribacter.s.splanchnicus | 0,2642 | 0,3164 | 0,2771 | 0,2771 |
| SV283.f.Ruminococcaceae.g.Butyricicoccus | 0,1175 | | 0,4355 | | 0,1006 | 0,2156 |  | SV401.o.Clostridiales.f.Ruminococcaceae | 0,1538 | 0,0196 | 0,0907 | 0,1089 |
| SV229.f.Ruminococcaceae.g.Ruminiclostridium_5 | 0,0849 | | 0,0943 | | 0,3937 | 0,4218 |  | SV214.f.Ruminococcaceae.g.Ruminiclostridium_5 | 0,3426 | 0,2058 | 0,0486 | 0,0729 |
| SV28.f.Alcaligenaceae.g.Parasutterella | 1,6327 | | 2,3960 | | 0,2113 | 0,2882 |  | SV7.g.Bacteroides.s.uniformis | 3,7100 | 2,1084 | 0,0380 | 0,0729 |
| SV24.g.Lachnospira.s.pectinoschiza | 0,6276 | | 1,1596 | | 0,0907 | 0,2156 |  |  |  |  |  |  |
| SV2.f.Bacteroidaceae.g.Bacteroides | 16,3367 | | 18,8141 | | 0,2113 | 0,2882 |  | pvalue < 0.1 |  |  |  |  |
| SV77.f.Lachnospiraceae.g.Lachnoclostridium | 2,7109 | | 1,9527 | | 0,1006 | 0,2156 |  | Adj. pvalue <0.1 |  |  |  |  |
| SV130.o.Clostridiales.f.Lachnospiraceae | 0,7165 | | 0,3491 | | 0,2113 | 0,2882 |  | 0.10 < Adj. pvalue < 0.11 |  |  |  |  |
| SV127.f.Ruminococcaceae.g.Oscillibacter | 0,3630 | | 0,2794 | | 0,2948 | 0,3685 |  |  |  |  |  |  |
| SV88.g.Coprococcus_3.s.comes | 1,1199 | | 0,6774 | | 0,1807 | 0,2882 |  |  |  |  |  |  |
| SV70.g.Anaerostipes.s.hadrus | 1,0572 | | 0,8430 | | 0,0502 | 0,2156 |  |  |  |  |  |  |
| SV32.g.Dorea.s.longicatena | 2,5283 | | 2,7906 | | 0,3711 | 0,4218 |  |  |  |  |  |  |
| SV93.f.Erysipelotrichaceae.g.Erysipelotrichaceae_UCG.003 | 1,2322 | | 0,6905 | | 0,0295 | 0,2156 |  |  |  |  |  |  |

Mann-Whitney test (panel A, B) and Wilcoxon test (panel from C to F) for mean relative abundances of the top 15 (A to E) and the top 6 (F) discriminant ASVs obtained by sPLS-DA. Benjamini-Hochberg multiple test correction procedure is performed to adjust p-values.

**Table S 5**

| **treatment** | **Mean values** | p-value |
| --- | --- | --- |
| BLM pMS T0 | 0.857 | 0.0537* |
| BLM pMS T1 | 0.428 |  |
| PBO pMS To | 1.5 | 0.6091 |
| PBO pMS T1 | 1.625 |  |
| BLM HBI T0 | 1.857 | 0.766 |
| BLM HBI T1 | 1.714 |  |
| PBO HBI T0 | 1.09 | 0.1661 |
| PBO HBI T1 | 0.90 |  |

Mean values for Partial Mayo Score( pMS) and Harvey-Bradshaw Index ( HBI) in patients untreated ( T0) and treated(T1) with Butyrose ( BLM) or placebo ( PBO) . * close to statistical significance

**Table S 6**

| FC ( ug/g) | BLM | | | PBO | | |
| --- | --- | --- | --- | --- | --- | --- |
|  | **CD T0** | **CD T1** | p-value | **CD T0** | **CD T1** | p-value |
| mean | 223.72 | 158.85 | 0.84 | 278.25 | 325 | 1 |
| (IQR) | 271 | 100 |  | 250.75 | 322 |  |
|  |  |  |  |  |  |  |
|  | **UC T0** | **UC T1** |  | **UC T0** | **UC T1** |  |
| mean | 187.93 | 214.21 | 0.35 | 684.57 | 304 | 0.09 |
| (IQR) | 166 | 231.5 |  | 1220.5 | 264.7 |  |
|  |  |  |  |  |  |  |

Fecal calprotectin levels (mean and Interquartile range) for each group (CD, UC ), treatment (Butyrose, Placebo) and Timepoint (T0 or T1).
